# Performance of diagnostic assays used to detect *Cryptosporidium* oocysts in faecal samples of cattle in Kuwait and genotyping of *Cryptosporidium* species

**DOI:** 10.1186/s12917-022-03435-w

**Published:** 2022-09-07

**Authors:** Nadra-Elwgoud M. I. Abdou, Maha S. AlAzemi, Mohammed T. Al-Sayegh, Qais A. H. Majeed

**Affiliations:** 1 GCC-Early Warning Center, PAAFR, Postal code, 1307 Rabyia, Kuwait; 2 Department of Medicine and Infectious Diseases, Faculty of Veterinary Medicine, Cairo University, Post code 12211, Giza, Egypt; 3 Department of Science, College of Basic Education, PAAET, Post code 23167, Aridyia, Kuwait

**Keywords:** *Cryptosporidium* spp., mZN, Immunochromatography, ELISA, PCR, Cattle, Kuwait

## Abstract

**Backgroud:**

*Cryptosporidium* species are zoonotic protozoan parasites responsible for gastroenteritis in various animals and humans. The diagnosis of *Cryptosporidium* presents many challenges. This research attempted to match the diagnostic efficiency of the modified Ziehl–Neelsen technique (mZN), immunochromatographic assays (IC), and enzyme-linked immunosorbent assay (ELISA) for the detection of *Cryptosporidium* in faecal samples of cattle in Kuwait. In addition, polymerase chain reaction (PCR) was utilised to determine the predominant species infecting cattle in Kuwait and correlating the detected species with the results of different diagnostic tests used, the presence or absence of clinical signs, and the age group of the infected cattle.

**Results:**

Of 400 analysed faecal samples, *Cryptosporidium* positive samples were 23%, 15.25%, and 14% using IC, ELISA, and mZN. IC had the highest sensitivity (74.07%), and mZN had the highest specificity (98.29%) using a composite reference standard (CRS) as a gold standard. The rapid IC test results in high false-positive results of cryptosporidiosis, whereas using mZN alone is insufficient to declare a negative faecal sample. Only 74.5% (35/47) of *Cryptosporidium*-positive samples by the three assays could be amplified by PCR. This study was the first to genotype *Cryptosporidium* in Kuwait. *Cryptosporidium parvum* (*n* = 26) was the dominant species detected from cattle samples, followed by *C. andersoni* (*n* = 6), *C. bovis* (n = 2), and *C. raynae* (*n* = 1). The findings showed a statistically relevant relationship between diarrhoea and the detection of *Cryptosporidium* spp. in faecal samples of cattle (*p*-value = 0.0003). Pre-weaned calves were the most vulnerable age group to *Cryptosporidium* spp. infection (*p*-value = 0.0007).

**Conclusion:**

For screening of *Cryptosporidium* infection in faecal samples, antigen detection or PCR methods combined with one of the microscopy techniques should be used. *Cryptosporidium parvum* was the prepoderant *Cryptosporidium* spp. recovered from cattle samples in Kuwait followed by *C. andersoni*. *Cryptosporidium parvum* is a significant risk factor for diarrhoea in pre-weaned calves. However, further study is needed as many other causes of diarrhoea in calves must be ruled out before a diagnosis of *Cryptosporidium* diarrhoea can be made.

**Supplementary Information:**

The online version contains supplementary material available at 10.1186/s12917-022-03435-w.

## Background

*Cryptosporidium* species, the enteric apicomplexan protozoan parasite, can infect various hosts, including humans, livestock and poultry. However, cattle are the most significant host from a veterinary and public health perspective [1]. However, ten *Cryptosporidium* spp. have already been recorded in cattle, only four spp.; *Cryptosporidium parvum*, *C. bovis*, *C. ryanae*, and *C. andersoni* are the most frequently found [2–5]. Nevertheless, *C. parvum* is the only spp. linked to clinical illness in neonatal calves, with older animals (> 6 weeks) demonstrating asymptomatic oocyst shedding [3]. *Cryptosporidium bovis* and *C. ryanae* are the predominant species in post-weaned calves [3–5]. There is little evidence on the clinical symptoms caused by *C. bovis* and *C. ryanae*; although, diarrhoea was reported in native calves in Nigeria, presumably due to *C. bovis* and *C. ryanae* infection [6]. *Cryptosporidium andersoni* is more commonly found in adults than in young cattle. *Cryptosporidium andersoni* infections are clinically associated with weight gain impairment and reduced milk yield in adult cows [2, 7].

Diagnosis of *Cryptosporidium* spp. in clinical cases, the specimen typically contains considerable oocysts count and a high concentration of the parasite antigen, even though methods with low sensitivity grant a positive result. Whereas specimens with few oocysts may necessitate an epidemiological investigation to detect asymptomatic carriers, using an initial screening method then a confirmatory test, for example, molecular or microscopic techniques, can boost reliance in the diagnosis [8]. Furthermore, accurate and rapid detection of *Cryptosporidium* spp. during diarrhoea epidemics in calves can help perform timely interventions, reduce economic losses, and improve animal welfare [9].

Several diagnostic assays were applied to detect Cryptosporidiosis in various hosts. They include 1- conventional (faecal smears stained by mZN), 2- antigens-detection tests (IC and ELISA), and 3- detection of *Cryptosporidium* DNA (PCR). Conventional microscopy is time-consuming, laborious, and needs expert microscopists to identify oocysts accurately. Simultaneously ELISA and PCR are costly and require well-equipped laboratories and skilled technicians. Although IC is a rapid test and easy to perform and interpret but may have many false-positive results [10, 11].

Many diagnostic circumstances lack a gold standard, and it can be argued that what is popularly known as a gold standard may not be a proper one. As a result, numerous approaches to assessing diagnostic tests in the lack of a gold standard have been established [12, 13]. For instance, a composite reference standard (CRS) can be created by combining the results of numerous imperfect tests, excluding the index test (the test to be evaluated). Based on a predetermined rule, a CRS is thought to have better discriminatory qualities than each individual reference standard [14]. Additionally, when more than two reference tests are included in the composite reference standard, the final definition of the disease may become muddled [15]. The exclusion of the index test from the composite reference standard is essential to avoid incorporation bias [16].

All assays used commonly in Kuwait for detecting clinical and asymptomatic cryptosporidiosis in cattle have not been evaluated. Thus, the objectives of this study were to assess routinely used laboratory tests such as microscopic examination of mZN stained faecal smears, IC, and ELISA. Given that there is no gold standard assay for diagnosis of Cryptosporidiosis [8], we applied a composite reference standard to create a pseudo-gold standard for evaluating the tests used to detect *Cryptosporidium* oocysts. Genotyping of *Cryptosporidium* species was applied. In addition, correlating the detected species with the results of different diagnostic tests used, the presence or absence of clinical signs, and the age group of the infected cattle were also studied.

## Results

The total number of cattle on the visited farms was 9365. Rectal faecal samples were collected randomly from 400 cattle: 175 pre-weaned, 49 post-weaned, and 176 adults.

### Performance of mZN, IC, and ELISA in diagnosis of *Cryptosporidium*

Examination of 400 cattle faecal samples for *Cryptosporidium* oocysts and antigens revealed that the *Cryptosporidium* positive samples were 23%, 15.25%, and 14% using IC, ELISA, and mZN, respectively (Table. 1). Results of the three diagnostic tests used showed that 287 (71.75%) faecal samples were negative, whereas 33 (8.25%) faecal samples were positive using the three tests (Table 1). Different distribution of *Cryptosporidium* results according to the examined test used, mZN, IC, ELISA, in faecal samples of cattle were recorded in Table 1. Concerning other entero-pathogens discovered by the IC test, rotavirus, coronavirus, and *E. coli* were found in 21 (5.25%), 4 (1.0%), and 73 (18.25%), respectively. In addition, coinfections of rotavirus with *Cryptosporidium* spp. and *E. coli* with *Cryptosporidium* spp. were detected in 2.25% (9/400) and 4.5% (18/400), respectively. Mixed infections with *Cryptosporidium* spp., rotavirus, and *E. coli* were detected in two of 400 examined faecal samples (0.5%).

**Table 1:** Distribution of *Cryptosporidium* results according to the test used; mZN, IC, ELISA, in faecal samples of cattle (*N* = 400)

mZN/IC/ELISA	Observed frequency (n)	Observed proportion (n/N)
+ / + / +	33	8.25%
+/-/-	5	1.25%
+/+/-	15	3.75%
+/-/+	3	0.75%
-/ + / +	12	3.00%
-/+/-	32	8.00%
-/-/ +	13	3.25%
-/-/-	287	71.75%
Total number of positive results		
mZN	56	14.00%
IC	92	23.00%
ELISA	61	15.25%

The diagnostic sensitivity of mZN, IC, and ELISA to detect *Cryptosporidium* in cattle faeces was 47.22%, 74.07%, and 48.00%, respectively. In contrast, the diagnostic specificity was 98.29%, 89.97%, and 95.67% for mZN, IC, and ELISA, respectively. The agreement between IC and the pseudo-gold standard was substantial (κ = 0.61). On the other hand, a moderate agreement was reported for both mZN and ELISA compared to the pseudo-gold standard (κ = 0.54 and 0.50, respectively). Table 2 summarises the diagnostic performance meassures of the three test, including accuracy, Positive Predictive Value (PPV), and Negative Predictive Value (NPV).

**Table 2:** Performance of the three test (mZN, IC, ELISA) used to detect *Cryptosporidium* in feces of cattle compared with the CRS

	mZN	IC	ELISA
True positive	51	60	48
False positive	5	32	13
True negative	332	287	287
False negative	12	21	52
Sensitivity (**95% CI**)	47.22% (37.54% to 57.06%)	74.07% (63.14% to 83.18%)	48.00% (37.90% to 58.22%)
Specificity (**95% CI)**	98.29% (96.05% to 99.44%)	89.97% (86.13% to 93.04%)	95.67% (92.70% to 97.67%)
PPV (**95% CI)**	91.07% (80.70% to 96.14%)	65.22% (56.85% to 72.74%)	78.69% (67.63% to 86.71%)
NPV (**95% CI)**	83.43% (80.81% to 85.76%)	93.18% (90.42% to 95.19%)	84.66% (82.03% to 86.97%)
Accuracy (**95% CI)**	84.50% 80.57% to 87.91%)	86.75% (83.03% to 89.91%)	83.75% (79.76% to 87.23%)
Cohen’s Kappa Test*	0.54	0.61	0.50
	Moderate agreement	Substantial agreement	Moderate agreement

^*^ The interpretation of *κ*-values: no agreement (*κ* =  < 0), slight (*κ* = 0.00–0.20), fair (*κ* = 0.21–0.40), moderate (*κ* = 0.41–0.60), substantial (*κ* = 0.61–0.80), and perfect (*κ* = 0.81–1.00)

### Comparison between the three diagnostic tests with the PCR results

A comparison between the three diagnostic tests used to detect *Cryptosporidium* with the PCR results and the genotype identified in 47 faecal samples was reported in Table 3. Out of 47 faecal samples, 35 samples (74.5%) were positive by nested PCR for detection of *Cryptosporidium* species. Thirty-two of the 35 positive PCR samples gave positive result by IC (91.4%), 28 samples were positive by mZN (80.0%), and 24 samples were positive by ELISA (68.6%). *Cryptosporidium parvum* was detected in 26 samples; 15 samples were positive by mZN, IC and ELISA, six were positive by IC and ELISA, and five were positive by mZN and IC. *Cryptosporidium andersoni* was detected in six samples; two were positive by both mZN and IC, whereas two were positive by both mZN, and ELISA. The other two samples were positive only by one test, mZN or IC (Table 3). Twelve faecal samples were negative by PCR; 100% (12/12) were positive by IC, 75% (9/12) were positive by mZN, and 66.6% (8/12) were positive by ELISA (Table 3).

**Table 3:** Comparison of positive results of different diagnostic tests used for the detection of *Cryptosporidium* with the PCR results and the genotype identified in the examined 47 faecal samples

PCR + ve (*n* = 35)				
mZN	IC	ELISA	Genotype	Total number
+	+	+	*C. parvum*	15
-	+	+	*C. parvum*	6
+	+	-	*C. parvum*	5
+	+	-	*C. andersoni*	2
+	-	+	*C. andersoni*	2
+	-	-	*C. andersoni*	1
-	+	-	*C. andersoni*	1
+	+	-	*C. bovis*	1
+	+	+	*C. bovis*	1
+	+	-	*C. ryanae*	1
28/35 (80.0)	32/35 (91.4)	24/35 (68.6)	*Total (%)*	
PCR -ve (*n *= 12)				
mZN	IC	ELISA	Genotype	Total number
-	+	-	-	2
+	+	-	-	2
-	+	+	-	1
+	+	+	-	7
9/12 (75.0)	12/12(100)	8/12 (66.6)	*Total (%)*	

### *Cryptosporidium* spp. association with diarrhoea and age groups

Statistical correlation between faecal consistency, age group, and different genotypes of *Cryptosporidium* spp. showed that *C. parvum* was recorded in 46.8% (22/47) diarrheic pre-weaned cattle and four adult cattle (8.5%) with normal faecal consistency (Table 4). Whereas *C. andersoni* was detected in four adult cattle (8.5%) with normal faecal consistency, one pre-weaned cattle with normal faecal consistency, and one diarrheic post-weaned cattle. *C. bovis* was detected in two pre-weaned cattle with normal faecal consistency, and one *C. ryanae* was diagnosed in post-weaned cattle with normal faecal consistency (Table 4). Diarrhoea and the diagnosis of *Cryptosporidium* spp. had a statistically strong relationship (*p-*value = 0.0003). Pre-weaned calves were the age group most likely to contract *Cryptosporidium* spp. infection (*p-*value = 0.0007).

**Table 4:** Statistical correlation between PCR results and the different *Cryptosporidium* spp. with faecal consistency, and age group in the examined cattle (47 faecal samples)

PCR result	Identified genotype	Age group	Faecal consistency	Total + ve animals (%)
*Positive*	*C. andersoni*	Adult	Normal	4 (8.5)
	*C. andersoni*	Post-weaned	Diarrhoea	1 (2.1)
	*C. andersoni*	Pre-weaned	Normal	1 (2.1)
	*C. bovis*	Pre-weaned	Normal	2 (4.3)
	*C. ryanae*	Post-weaned	Normal	1 (2.1)
	*C. parvum*	Adult	Normal	4 (8.5)
	*C. parvum*	Pre-weaned	Diarrhoea	22 (46.8)
*Negative*	*-*	Adult	Diarrhoea	4 (8.5)
	*-*	Post-weaned	Normal	1 (2.1)
	*-*	Pre-weaned	Diarrhoea	7 (15.0)
*p-*value		0.0007	0.0003	47 (100)

Pre-weaned < 3 months, Post-weaned 3–24 months, Adult  ≥24 months; full milk teeth. Normal faecal consistency (formed or firm but not hard), diarrhoea (runny, watery, liquid consistency)

### Phylogenetic analysis of detected *Cryptosporidium* species

**Fig. 1:**
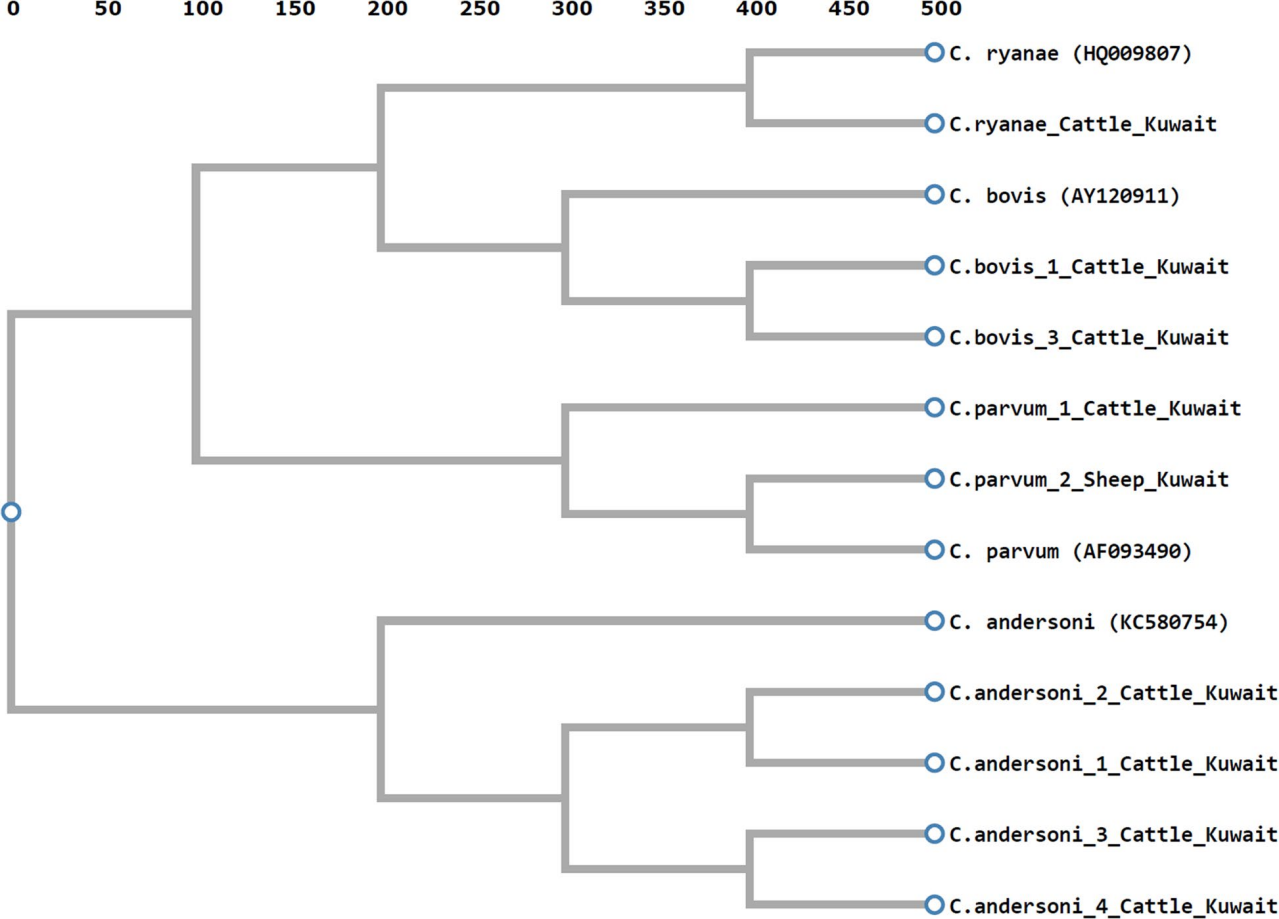
Phylogenetic sequence relationship of recovered *Cryptosporidium* spp. to other species in Genbank; *C. andersoni* isolated from cattle in China (KC580754), *C. parvum*; strain Bovine *C. parvum* genotype (BOH6) isolated from calf in Ohio (AF093490), *C. bovis*; Bovis 2622 isolated from cattle (AY120911), *C. ryanae* isolated from calves in China (HQ009807), and *C. parvum_*2*_*Sheep_Kuwait isolated from sheep in Kuwait

The phylogenetic tree shows that the current strains isolated from cattle clustered based on the species of C*ryptosporidium*, forming 4 groups (Ryanae, Bovis, Parvum, and Andersoni). Among the same species of *Cryptosporidium*, subgroups were formed like in the *C. andersoni* group. This indicates genetic variation within the genus *Cryptosporidium* (Fig. 1).

## Discussion

The detection and diagnosis of *Cryptosporidium* present many challenges. The presence of oocysts (itself, antigen, or DNA) is routinely used to detect *Cryptosporidium* infection in faecal samples using various laboratory techniques [17]. Because of the variety of diagnostic procedures used and the inconsistent use of typing methodologies, direct comparisons between clinical, veterinary, and environmental tests can be difficult or, indeed, impossible [17].

Previous studies evaluated different diagnostic techniques applied to identify *Cryptosporidium* oocysts in faecal samples of various animals using a variety of gold standards such as cumulative positivity [18] and latent class analysis model [10]. However, Danišová et al. [19] considered PCR the gold standard reference test because PCR has high accuracy, although PCR results could be affected by the presence of low-density oocysts in the faecal samples that may contain PCR inhibitors [20]. Additionally, the preservatives may penetrate the oocysts, which cannot be removed by washing, consequently inhibiting PCR results [17]. Furthermore, PCR techniques are expensive and need specialized equipment, which is not available in every laboratory. However, the main advantage of using PCR is identifying the infecting species since most diagnostic assays only detect the presence or absence of *Cryptosporidium* oocysts. Identifying *Cryptosporidium* species is significant because different species have different epidemiology, clinical manifestations, and sequelae [1].

In the present study, the performance of the three tests (mZN, IC, ELISA) used to detect *Cryptosporidium* in cattle faeces was studied. The results revealed that IC had the highest sensitivity, and mZN had the highest specificity using a CRS as a gold standard. The sensitivity of the three tests ranged from low in mZN and ELISA (47.22%, 48%, respectively) to moderate in the IC test (74%), whereas the specificity of the three tests was high (90% or higher). The low sensitivity of the three tests may be due to the low intensity of infection rate in the studied cattle population, and the present study was a cross-sectional study where only one faecal sample was collected from each animal. However, repeated examination of more than one faecal sample on three consecutive days enhances the detection of *Cryptosporidium* oocysts [21].

Ezzaty Mirhashemi et al. [10] found that ELISA and PCR had higher specificity than standard microscopic assay (Kinyoun’s carbol-fuchsin acid-fast staining) in cattle samples. While utilizing LCA as the gold standard, routine microscopic analysis in sheep samples exhibited the highest specificity when compared to ELISA and PCR [10]. A previous study evaluated different diagnostic methods used to detect *Cryptosporidium* in stools of diarrheic children using PCR as a gold standard. mZN revealed higher specificity than ELISA and rapid strip, while ELISA showed the highest sensitivity [22]. In another study, using PCR as a gold standard to evaluate immunological tests for diagnosis of *Cryptosporidium* in diarrheic animals (pigs, calves, and lambs), ELISA (40.9%) showed higher sensitivity than IC (22.7%), while IC (100%) showed higher specificity than ELISA (78.9%) [19]. Papini et al. [18] studied the performance of three IC tests to detect *C. parvum* in diarrheic calves using cumulative positivity as a gold standard. They found that the three kits had high sensitivity and specificity (SE 100%, 100%, and 90.24%, SP 96%, 92%, 100%, respectively).

Different sensitivities and specificities of diagnostic tests used to diagnose *Cryptosporidium* in faecal samples have been reported. These differences are highly dependent on infecting species and the concentration of oocysts in the faecal sample [10, 23].

The diagnostic performance of mZN revealed a high PPV and NPV; such a higher value may be because mZN confirms the diagnosis by tracing the parasites. It was reported that the microscopic method for detection of *Cryptosporidium* spp. in faeces is highly specific and less sensitive [24] and can be used as a confirmatory test. However, there is a great demand for an alternative test to overcome the limitations of this assay, such as the lack of experts microscopist for accurate identification of oocysts [25].

Detecting *Cryptosporidium* antigens (copro-antigens) in faecal samples using ELISA and IC assays is widely used. However, ELISA and IC detection limits ranged from 3 × 10^5^ to 10^6^ oocysts per ml [8, 26], which is not significantly more sensitive than conventional microscopy. In comparison to microscopy, ELISA, and PCR procedures, the rapid IC assay was quicker and simpler to do because it didn't call for any extra special equipment. This reaerch reported false-positive results with rapid IC tests consistent with previous studies [22, 27]. The IC test had a PPV of 65.22%, indicating that positive results with this assay need to be confirmed microscopically or using PCR. Hence, rapid IC kits can be used as a screening test during diarrhoea outbreaks.

In the present study, the results of immunological tests used to detect different *Cryptosporidium* spp. were controversial. One possible explanation is that not all commercially produced antibodies recognize all *Cryptosporidium* spp. oocyst antigens of individual species. Hence, immunological testing cannot detect every copro-antigen found in the more than 30 species and genotypes identified until 2016 [19].

Only 74.5% (35/47) of *Cryptosporidium*-positive samples recovered by the three assays could be identified by PCR. This could be attributable to different factors, including the low PPV of IC and ELISA or PCR inhibition. *Cryptosporidium parvum* was the most common species detected from cattle samples in Kuwait, with only a few samples containing *C. andersoni* (*n* = 6), *C. bovis* (*n* = 2), and *C. raynae* (*n* = 1). There have been few studies of *Cryptosporidium* molecular diagnosis in the Middle East. In terms of *Cryptosporidium* spp. distribution, our results differ from those of previous studies. *Cryptosporidium parvum* was the only discovered species in Tunisia and Syria [28–30]. In contrast, other studies reported *C. parvum and* other *Cryptosporidium* spp. infecting cattle farms. *Cryptosporidium parvum* was recorded as the most predominant species, with *C. andersoni* in Iran [31], *C. raynae* in Turkey [32], *C. bovis* and *C. raynae* in Egypt [33], and *C. ryanae*, *C. andersoni,* and *C. bovis* in Sudan [34]. The most prevalent *Cryptosporidium* identified in Jordan was *C. ryanae,* then *C. parvum* and *C. andersoni* [35]. In Algeria, *C. bovis* was the most detected spp. in cattle, then *C. ryanae* and *C. parvum* [36]. The four *Cryptosporidium* spp. that infect cattle are distributed differently over the world [1].

In this study, *C. andersoni* was detected in six samples from four adult cattle, one post-weaned and one pre-weaned. All positive animals were from the same farm. Only one animal had diarrhoea (post-weaned), also was positive for both rotavirus and *E. coli* by rapid IC test. Another positive animal was a cow with a history of decreased milk production and severely emaciated. *Cryptosporidium andersoni* was identified in adult cattle more than in young cattle [37]. In addition, infections with *C. andersoni* have been linked to decreased weight gain and milk production in adult cows [7]. Whereas *C. bovis* was reported in two healthy pre-weaned calves, and *C. ryanae* was identified from one healthy post-weaned animal. Previous studies reported that neither *C. bovis* nor *C. ryanae* have been implicated in clinical disease in cattle of any age group [4, 38–40].

In the present research, *C. parvum* was the prepoderant species detected from cattle in Kuwait. It was identified in four adult cattle without signs of diarrhoea, and 22 pre-weaned calves suffered from diarrhoea. *C. parvum* is a widely endemic pathogen that causes self-limiting gastroenteritis in pre-weaned calves suffered from profuse watery diarrhoea as a common symptom and can be fatal in severe cases [41, 42]. Multiple pathogens (rotavirus, coronavirus, pathogenic strains of *Escherichia coli*, and *Salmonella* spp.) can cause neonatal diarrhoea [41]. In contrast, cryptosporidiosis is confirmed as a major diarrhoeal cause in pre-weaned calves by detecting significant numbers of oocysts in the absence of other pathogens; however, coinfection has been commonly believed to alter the clinical presentation and lead to more severe cryptosporidiosis [42].

The detected *Cryptosporidium* spp. were clustered into four groups (Ryanae, Bovis, Parvum, and Andersoni) in the phylogenetic tree. In addition, subgroups of *Cryptosporidium* were formed among the same species. This indicates genetic variation within the genus *Cryptosporidium* [43].

## Conclusions

The antigen detection test results in false-positive results of cryptosporidiosis, whereas using mZN alone is insufficient to declare a negative faecal sample. As a result, for screening of *Cryptosporidium* infection in faecal samples, antigen detection or PCR methods combined with one of the microscopy techniques should be used. *Cryptosporidium parvum* is a significant risk factor for diarrhoea in pre-weaned calves. However, further study is needed as many other causes of diarrhoea in calves must be ruled out before a diagnosis of *Cryptosporidium* diarrhoea can be made.

## Methods

### Study design

Between October 2014 to September 2015, a 1-year cross-sectional study was carried out to assess the diagnostic performance of several tests used to identify *Cryptosporidium* infections in cattle with or without clinical symptoms. The farms were chosen without being aware of the level of *Cryptosporidium* infestation. Twenty-two dairy cattle farms were visited once in the Sulaibiya area.

### Sample collection

Four hundred cattle were randomly selected to examine their faecal samples. Five to ten grams of faeces were collected from the rectum or immediately after defecation and kept in a sterile capped cup. The faecal samples were categorised according to their consistency into diarrhoeic (*n* = 127) and non-diarrhoeic (*n* = 273).

### Processing of samples

Each sample was split into three parts in the lab: the first part was used to identify *Cryptosporidium* oocysts using mZN; the second part was utilised to identify *Cryptosporidium* antigens using IC and ELISA; and the third part was stored either in 2.5% potassium dichromate or at -20 °C. If the sample was determined to be positive for a *Cryptosporidium* oocyst or antigen by mZN, IC, and/or ELISA, the stored sample was sent to Prof. Dr. Lihua Xiao for typing and subtyping of *Cryptosporidium spp.*

### Detection of *Cryptosporidium* oocyst

**Fig. 2:**
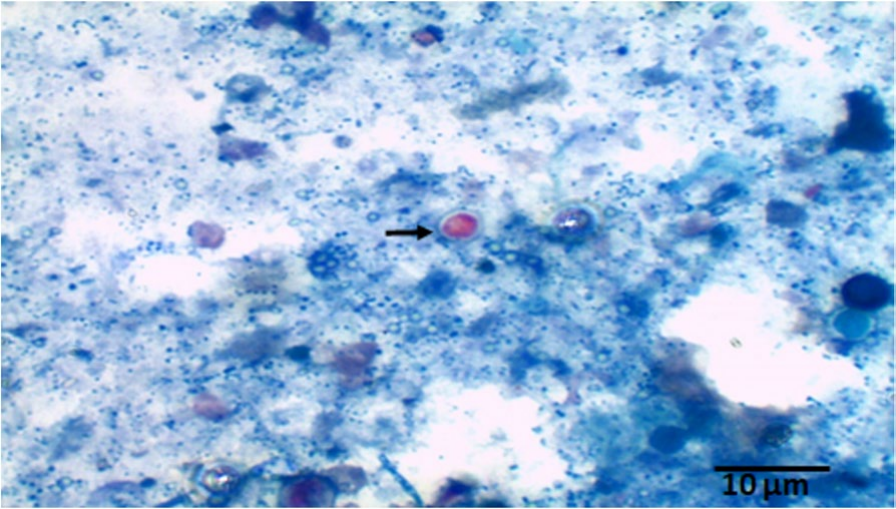
A mZN stained faecal smear, *Cryptosporidium* oocyst (arrow) pink spherical body against purple background

*Cryptosporidium* oocysts were identified conventionally using faecal smears stained with mZN (Fig. 2). Fresh and concentrated faecal smears were prepared, stained, and examined as formerly described [44]. Concisely, faecal smears were prepared on a microscope slide, air-dried at room temperature, then fixed for 5 min with pure alcohol (methanol). Fixed smears were stained for 3–5 min with dilute carbol-fuchsin (1: 10) before rinsing using water. Decolourisation step for 10–15 min with 3% HCL in ethanol, then counterstained for one minute with 0.5% malachite green solution. Finally, Smear slides were washed with tap water, air dried, and examined at × 400 magnification under the light microscope. Oocysts of *Cryptosporidium* spp. appear as pink to red bodies that are spherical to ovoid against a green to purple background. Samples were considered positive if at least one morphologically distinct *Cryptosporidium* species.

### Detection of *Cryptosporidium* antigen

**Fig. 3:**
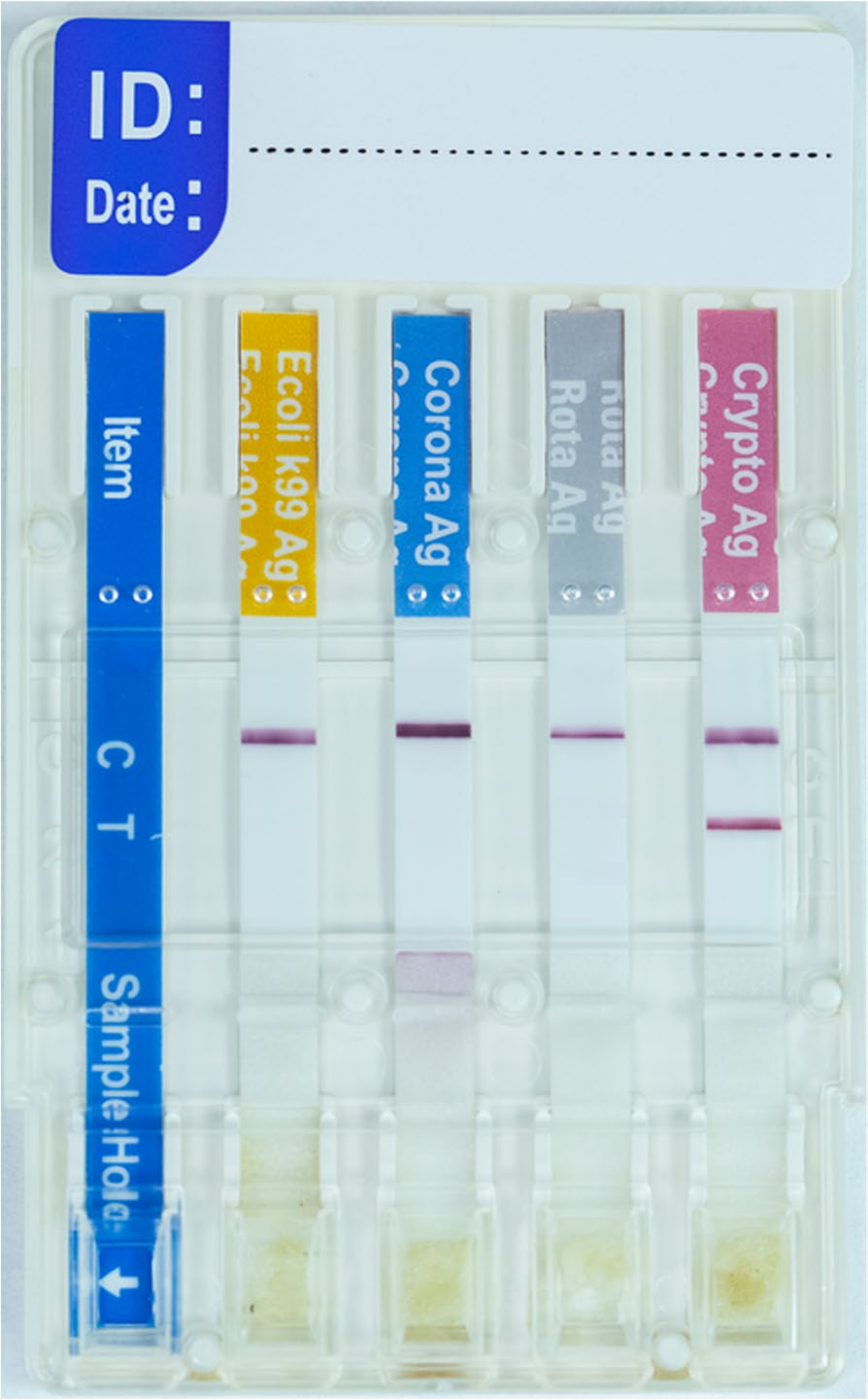
Bovid-4 kit device showed a positive result for *Cryptosporidium* antigen and negative for Rotavirus, coronavirus, and *E. coli* antigens. Diluted sample added into the sample hole, the results were interpreted after 5–10 min; the sample was considered negative if only the control line “C” appeared and positive if both “C” and “T” lines appeared

Rapid BoviD-4 Ag immunochromatography test kit (BioNote Inc., Gyeonggi-do, Korea) was utilised to discover *Cryptosporidium* spp., rotavirus, coronavirus, and *E. coli* K99 antigens in faeces. The right quantity of faeces was collected using a sterile swab stick. The swab placed into the dilution tube and left till fully dissolved in the diluent, then vigorously stirred. The tube was then covered and allowed for sedimentation for 30 min. One drop of supernatant was dispensed into the sample holes of the BoviD-4 Ag testing apparatus using a disposable dropper. The findings were interpreted in accordance with the manufacturer's recommendations (Fig. 3).

**Fig. 4:**
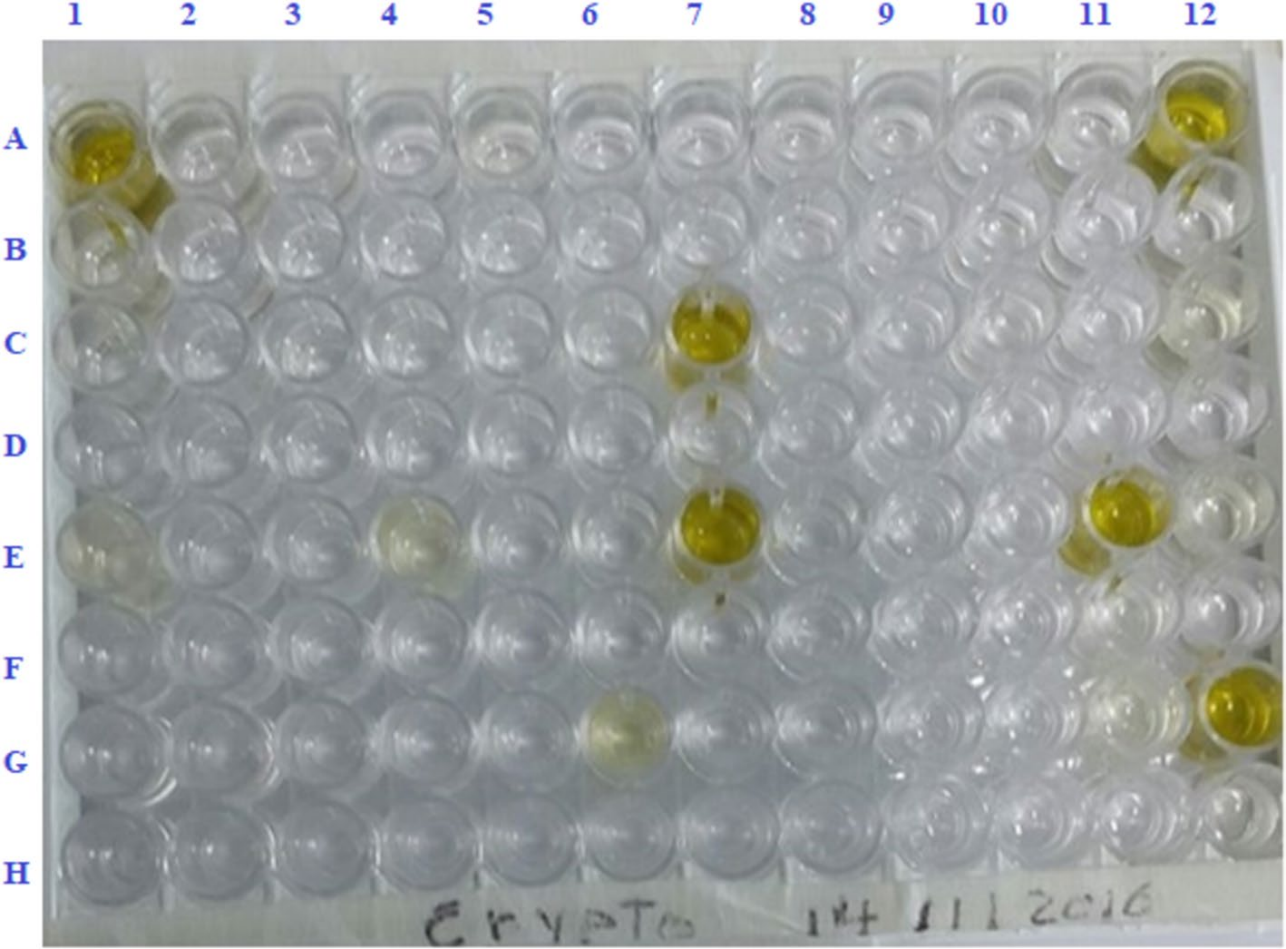
A sandwich, double wells ELISA plate showed positive samples for *Cryptosporidium* antigen. Rows A, C, E, G were sensitized with the anti-*Cryptosporidium*-specific antibody and rows B, D, F, H coated nonspecific antibodies. Control positive added in wells A1 and B1. The diluted samples added to the wells as follows: sample 1 in wells C1 and D1, sample 2 in wells E1 and F1, sample 3 G1 and H1, etc., till sample 47 in wells G12 and H12. Validation of the test; positive control optical density net (Delta) value < 1.151. Positive samples were numbers 25, 26, 42, 44 and 47 their S/P% (Delta optical density of sample/ Delta optical density of positive control *100) were > 7%

A sandwich, double wells ELISA kit (Bio-X Diagnostics, Rochefort, Belgium) was used to detect *Cryptosporidium* antigens. The plate is coated with the anti-*Cryptosporidium*-specific antibody. The methodology, and interpretation of the results were performed according to the manufacturer’s guidelines. Samples were stored at -20^*◦*^C without preservatives and the test was applied within a month from the day of collection. Faecal samples were diluted and added to the coated wells, then the plate was incubated for 1 h at 21^*◦*^ ± 3^*◦*^C. Next, the conjugated anti-*Cryptosporidium* monoclonal antibody was appended and reincubated. Finally, tetramethylbenzidine (TMB) was visualized the reaction as showed in Fig. 4, and the results were measured at 450 nm using a microplate reader (BioTeck ELX800G reader, BioTeck Instruments Inc., Winooski, USA).

### Extraction of *Cryptosporidium* DNA

Forty-seven samples were selected for PCR testing and molecular typing of *Cryptosporidium* species. Criteria of selection of samples for PCR test was mainly those positive by two or more methods except four samples were positive using one test (3 samples from cattle suffered from profuse watery diarrhoea and were positive only using IC, whereas one sample was positive only using mZN from a cow with a history of decreased milk production and severely emaciated). The samples preserved in potassium dichromate were centrifugated twice in distilled water. DNA was extracted from all specimens (frozen and preserved) using the FastDNA SPIN kit for soil (MP Biomedicals, Santa Ana, CA, USA).

### Nested PCR and RF LP of restriction enzymes *Ssp*I and *Mbo*II

**Fig. 5:**
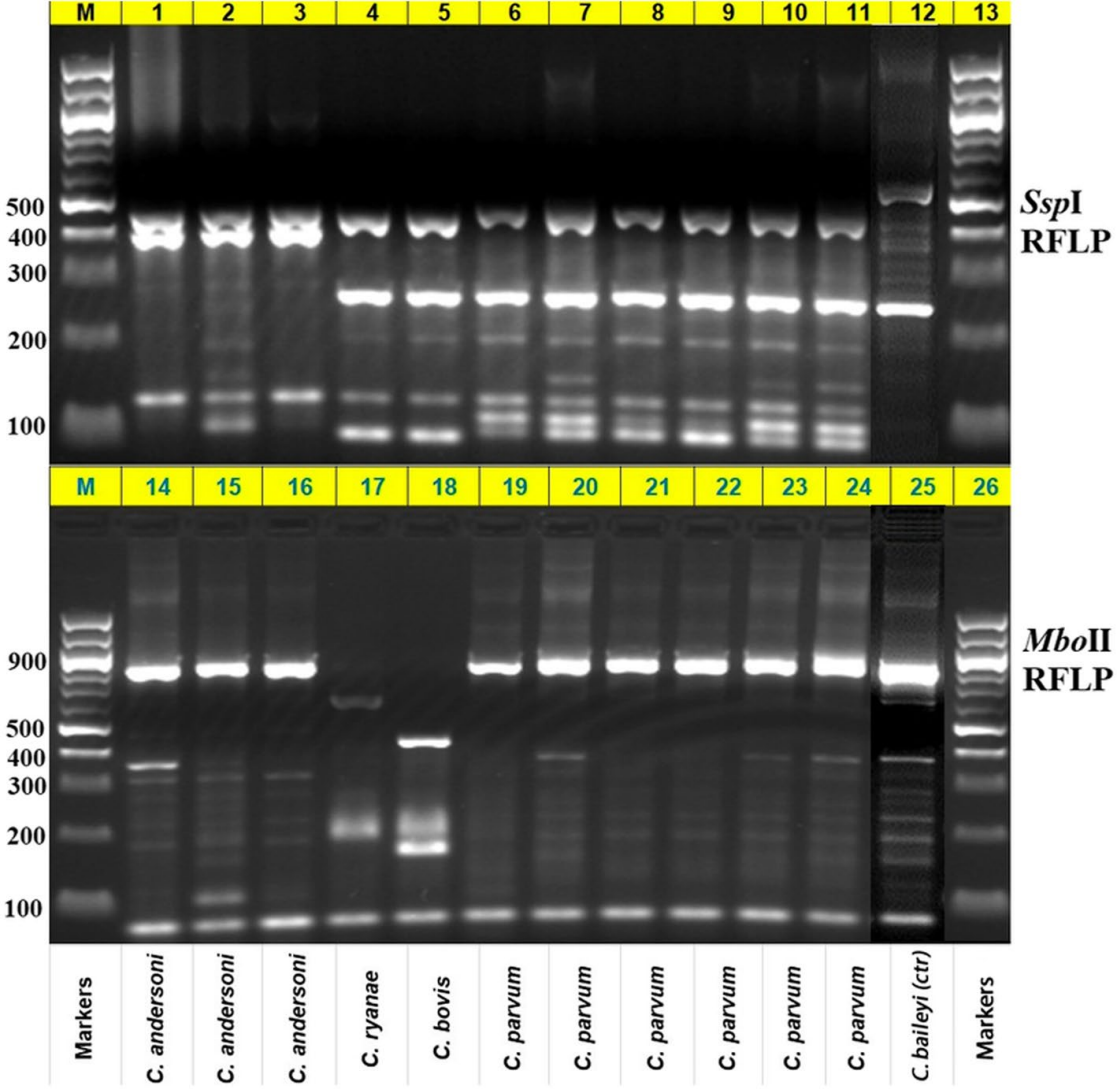
RFLP analysis identifying the four species of *Cryptosporidium* isolated from cattle samples. M: Markers; 100-bp molecular markers. Lanes 1–3: *C. andersoni Ssp*I products 448, 397 bp, and lanes 14–16: *C. andersoni Mbo*II products 769, 76 bp. Lane 4: *C. ryanae Ssp*I products 432, 267,103, 33 bp, and lane 17: *C. ryanae Mbo*II products 574, 185, 76 bp. Lane 5: *C. bovis Ssp*I products 432, 267,103, 33 bp, and lane18: *C. bovis Mbo*II products 412, 185, 162, 76 bp. Lanes 6–11 *C. parvum Ssp*I products 449, 267, 397, 12, 11 bp, and lanes 19–24: *C. parvum Mbo*II products 771, 76 bp. Lanes 12 and 25: *C. baileyi* (control sample)

Using a nested PCR targeting an approximately 830-bp fragment length of small subunit (SSU) rRNA gene was applied [45]. The primers utilized and the thermocycler program as formerly described [46, 47] are summarised in Supplementary file 1. *Cryptosporidium* molecularly differentiated by RFLP of the secondary PCR products using *Ssp*I and *Mbo*II (Fig. 5) as previously described [45]. Sequence analysis of the 60 KDa glycoprotein (gp60) gene was used to further analyze samples that tested positive for C. parvum at the SSU rRNA locus [47, 48].

### Phylogenetic analysis

The 18S rRNA gene sequences of the recovered *Cryptosporidium* spp. were aligned with publicly available sequences to determine the relationship between *Cryptosporidium* species using ClustalX (http://www.Clustal.Org/). The 18S rRNA sequences of *C. andersoni* isolated from cattle in China (KC580754), *C. parvum*; strain Bovine *C. parvum* genotype (BOH6) isolated from calf in Ohio (AF093490), *C. bovis*; Bovis 2622 isolated from cattle (AY120911), *C. ryanae* RYanaeS6293a1 isolated from calves in China (HQ009807), and *C. parvum_*2*_*Sheep_Kuwait isolated from sheep in Kuwait [49].

### Statistical analysis

A composite reference standard was applied to build a pseudo-gold standard for evaluating mZN, IC, and ELISA used to detect *Cryptosporidium.* The animal was classified as *Cryptosporidium* spp.-infected if either one of the reference tests, excluding the index test, were positive. For instance, to evaluate IC (index test), results of mZN and ELISA were used to build the pseudo-gold standard. The diagnostic performances and the 95% confidence interval of the three tests was computed using MdCalc® Statistical software. Using QuickCalcs, GraphPad Software®, kappa (*κ*) test agreement was computed and assessed. The Chi-square test (χ^2^) was used to evaluate the correlation between the different *Cryptosporidium* spp. diagnosed by PCR with the presence or absence of diarrhoea, and age group in the examined 47 faecal samples.

## Supplementary Information

**Additional file1.** Nested PCR and RF LP of restriction enzymes* Ssp*I and *Mbo*II

## Data Availability

The datasets used and/or analysed during the current study are not publicly available due [The authors still working on data for preparation of another manuscript] but are available from the corresponding author on reasonable request.

## Abbreviations

*C*.: *Cryptosporidium*
Spp.: Species
mZN: Modified Ziehl–Neelsen technique
IC: Immunochromatography test
ELISA: Enzyme Linked Immunosorbent Assay
PCR: Polymerase Chain Reaction
RFLP: Restriction Fragment Length Polymorphism
PPV: Positive predictive value
NPV: Negative predictive value
CI: Confidence interval
*κ*-values: Kappa test agreement
χ^2^: Chi-square test
