# Supplementary material for: Performance of diagnostic assays used to detect Cryptosporidium oocysts in faecal samples of cattle in Kuwait and genotyping of Cryptosporidium species
Source: BMC Vet Res. 2022 Sep 7;18:336. doi: 10.1186/s12917-022-03435-w (PMC9449277; doi:10.1186/s12917-022-03435-w)
Supplement: Supplementary file 1 — Additional file1. Nested PCR and RF LP of restriction enzymes SspI and MboII [file 12917_2022_3435_MOESM1_ESM.docx]

**Supplementary file 1**

**Nested PCR and RF LP of restriction enzymes *Ssp*I and *Mbo*II**

Two sets of primers were used to amplify the full-length SSU rRNA gene from each sample. First stage primers PCR:

Forward (F1): 5’-TTCTAGAGCTAATACATGCG-3’

Reverse (R1): 5’-CCCATTTCCTTCGAAACAGGA-3’.

Second stage primers PCR:

Forward (F2): 5’-GGAAGGGTTGTATTTATTAGATAAAG-3’

Reverse (R2): 5’- CTCATAAGG TGCTGAAGGAGTA -3’.

Using a thermocycler, an initial denaturation step at 94°C for 5 minutes was followed by 35 cycles of denaturation at 94°C for 45 seconds, annealing at 55°C for 45 seconds, and extension at 72°C for 1 min. Subsequently, a final extension of incubation at 72°C for 10 min was also included [46,47]. *Cryptosporidium* species were diagnosed by restriction fragment length polymorphism (RFLP) analysis of the secondary PCR products using *Ssp*I and *Mbo*II (Fig. 5) as previously described [45]. Each sample was analysed at least twice using reagent water as the negative control and DNA of *Cryptosporidium baileyi* as the positive control. The identification of *Cryptosporidium* species was confirmed by sequence analysis of the secondary PCR products from representative specimens. *C.* *parvum*-positive samples at the SSU rRNA locus were further analysed using sequence analysis of the 60 KDa glycoprotein (gp60) gene [47, 48]. Positive secondary PCR products were sequenced directly on an ABI 3130 Genetic analyser (Applied Biosystems, Foster City, CA, USA). Bi-directional sequences obtained were assembled using the ChromasPro (version 1.5) software (<http://technelysium.com.au/?page_id=27>), and aligned with each other and referenced sequences of each gene downloaded from Gen Bank using ClustalX (<http://www.Clustal.Org/>) to determine *Cryptosporidium* species (based on SSU rRNA sequences).
